# The Role of Lead Shielding in Preventing Gamma Radiation-Induced Microleakage in Nanohybrid Composite Restorations: An In Vitro Study

**DOI:** 10.7759/cureus.79349

**Published:** 2025-02-20

**Authors:** Dolphi Bansal, Rajinder Bansal, Tapas K Dora, Manu Bansal, Devinder Singh, Rimple Gupta, Saurabh Gupta, Seema Gupta

**Affiliations:** 1 Department of Conservative Dentistry and Endodontics, Guru Nanak Dev Dental College and Research Institute, Sunam, IND; 2 Department of Radiation Oncology, Homi Bhabha Cancer Hospital, Sangrur, IND; 3 Department of Conservative Dentistry and Endodontics, All India Institute of Medical Sciences, Jammu, IND; 4 Department of Orthodontics, Kothiwal Dental College and Research Centre, Moradabad, IND

**Keywords:** gamma radiation, lead, microleakage, nanocomposites, shielding

## Abstract

Introduction: Marginal microleakage is a significant factor that contributes to the failure of cavity restorations, ultimately leading to recurrent caries. These failures are primarily attributed to the properties of the restorative materials, which are often influenced by various environmental factors. Gamma radiation has been shown to have deleterious effects not only on dental hard tissues but also on composite restorative materials, potentially increasing microleakage. A viable approach to mitigate these effects is the implementation of protective shielding for vulnerable structures during radiotherapy. This study aimed to evaluate the impact of gamma radiation on the marginal microleakage of nanohybrid composite restorations, specifically comparing the extent of microleakage in restorations subjected to radiation with and without lead shielding.

Materials and methods: An in vitro study was conducted using 45 freshly extracted premolars divided into three groups: group 1 (control), group 2 (radiated without lead shielding), and group 3 (radiated with lead shielding). Class V cavities were prepared and restored using nanohybrid composite resin. Gamma radiation was administered using a cobalt-60 machine at a total dose of 70 Gy for seven weeks. A lead shielding of 11-mm was placed in the collimator for group 3. Microleakage was evaluated using a dye penetration technique, and data were analyzed using the Kruskal-Wallis test followed by post-hoc Dunn’s test for pairwise comparisons.

Results: Significant differences were observed in microleakage between the groups using the Kruskal-Wallis test (p = 0.001); however, post-hoc analysis revealed no statistically significant difference between groups 2 and 3 (p > 0.05). This showed that lead shielding did not significantly reduce microleakage compared with no shielding. Group 1 exhibited the least microleakage, with a mean dye score of 0.87 ± 0.83. Group 3 showed moderate microleakage (mean score = 2.06 ± 0.50), while group 2 exhibited the most severe microleakage (mean score = 2.47 ± 0.51).

Conclusion: Gamma radiation adversely affected the marginal integrity of nanohybrid composite restorations, with the control group showing the best performance in terms of minimal microleakage. While lead shielding showed some reduction in microleakage, it was not statistically significant compared with the group without shielding. This highlights the need to optimize radiation shielding strategies to protect dental tissues in patients undergoing head and neck cancer treatment. Further in vivo studies are recommended to evaluate the long-term effects and refine clinical practices for radiation protection.

## Introduction

The longevity and clinical success of dental restorations depend on their ability to maintain an intact marginal seal, preventing microleakage that can compromise the tooth-restoration interface [1]. Marginal microleakage refers to the penetration of fluids, bacteria, and other oral contaminants between the restorative material and tooth structure, which can lead to secondary caries, pulpal irritation, long-term postoperative sensitivity, and eventual failure of restoration [2]. Despite advancements in dental materials, microleakage remains a significant concern, particularly in composite restorations.

Nanocomposite restorative materials have emerged as superior alternatives to conventional composite resins owing to their enhanced mechanical properties, superior polishability, and reduced polymerization shrinkage [3]. These materials incorporate nanoscale fillers, which improve their strength, wear resistance, and aesthetic quality [4]. However, their long-term clinical performance can be influenced by external factors, including exposure to ionizing radiation such as gamma radiation. Abaza et al. [5] conducted a study that evaluated the effects of gamma radiation on the microshear bond strength (MBS) of laser-prepared cavities restored with nanofilled restorative materials and concluded that gamma radiation did not adversely affect the MBS and nanoleakage of nanocomposites.

Gamma radiation is widely employed in medical treatment, particularly for cancer therapy [6]. Patients undergoing radiotherapy for head and neck malignancies are often exposed to significant doses of gamma radiation, which can adversely affect oral tissues, tooth structure, and restorative materials [7,8]. While extensive research has explored the effects of radiation on dental hard tissues, little is known about how gamma radiation affects contemporary restorative materials, particularly nanocomposites, under different protective conditions such as lead shielding [9,10].

Radiation-induced degradation of dental composites can occur because of changes in polymer cross-linking, increased water sorption, and alteration of filler-matrix interactions [11]. These changes may lead to increased marginal gap formation, reduced bond strength, and eventual failure of restoration [12,13]. Importantly, the use of protective measures, such as lead shields, during radiation therapy can mitigate these adverse effects [14,15]. However, the efficacy of such protection in preventing microleakage in the restored teeth remains unclear.

This study aimed to evaluate the impact of gamma radiation on the marginal microleakage of nanohybrid composite restorative material in teeth exposed to radiation with and without the use of lead shielding. By comparing microleakage in irradiated teeth shielded with lead to those without protection, this study sought to determine the effectiveness of lead shielding in preserving the integrity of nanohybrid composite restorations. The null hypothesis established in this study posited that no statistically significant differences would be observed across the groups.

## Materials and methods

Study type

**Figure 1:**
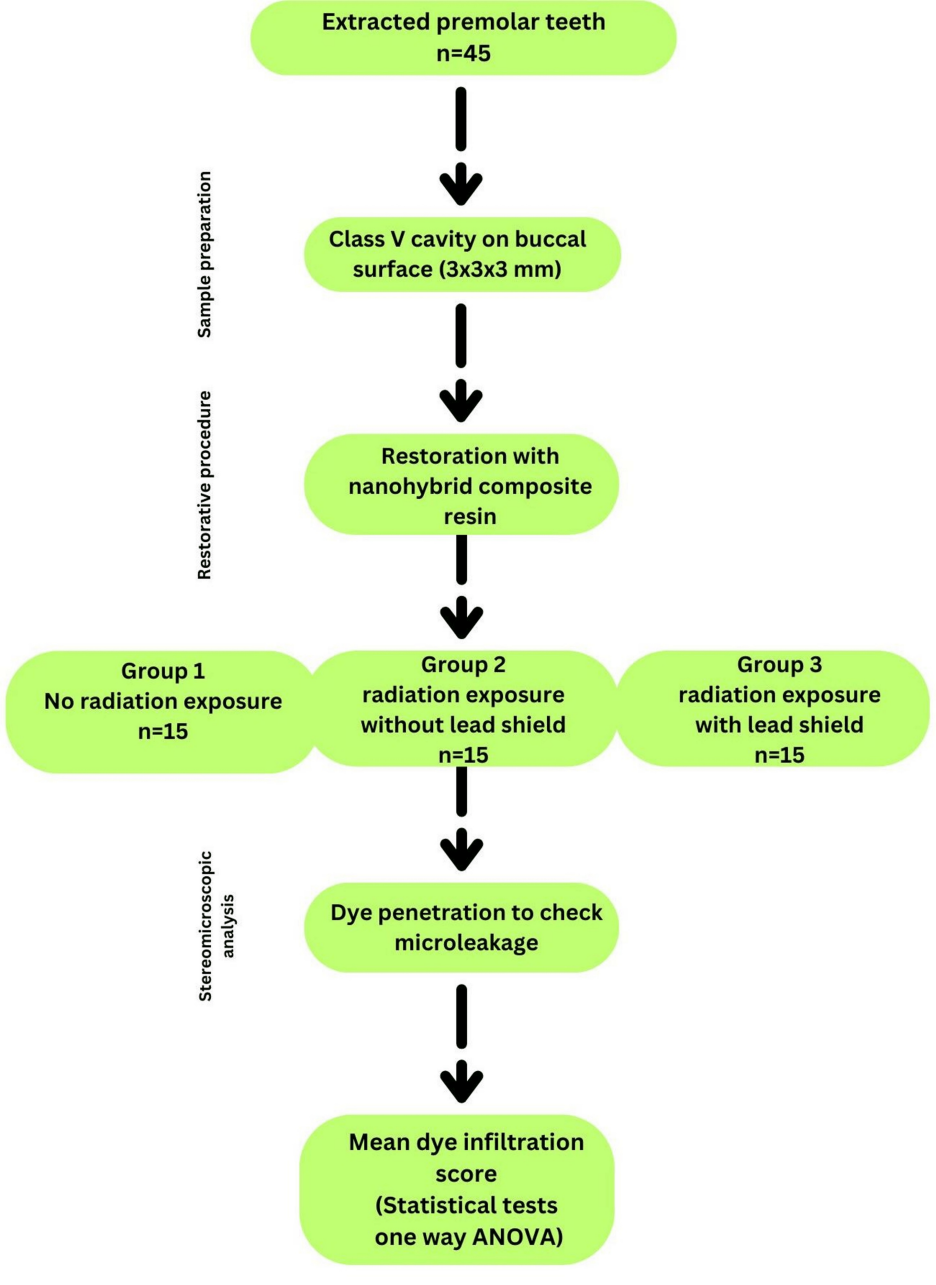
Flowchart illustrating the methodology of the study evaluating the effect of radiation exposure on microleakage.

This in vitro study was conducted in the Department of Conservative Dentistry and Endodontics at Guru Nanak Dev Dental College and Research Institute, Sunam between June 2023 and January 2024 (Figure 1).

The institutional ethical committee waived off the ethical approval, as it was an in vitro study in which extracted teeth were used with the consent of the patient. The study strictly adhered to the principles of the Declaration of Helsinki.

Sample size estimation

Sample size estimation was conducted using G*Power software version 3.1 (Heinrich Heine University Düsseldorf, Düsseldorf, Germany) to achieve a statistical power of 80%, with a significance level (alpha error) of 5%. Based on a minimum effect size of 0.5, derived from a prior study by Yoshikawa et al. [8], a total sample size of 45 teeth was determined to be adequate. The referenced study evaluated the marginal adaptation of various bulk-fill composites and reported a mean difference in dye infiltration of 0.2% with a pooled standard deviation of 0.4.

Sample collection

Non-cariogenic extracted premolars for orthodontic purposes were procured for the present investigation from the Department of Oral Surgery at Guru Nanak Dev Dental College and Research Institute, Sunam. Carious teeth, teeth with cracks, developmental defects, and fillings were excluded from this study. Debris and calculus present on the root surfaces were removed using a curette, preserved in distilled water that was changed on a weekly basis, and subsequently used within three months of the study duration. A total of 45 freshly extracted premolars were randomly allocated to three distinct groups.

Sample preparation

Class V cavities of consistent dimensions, precisely measuring 3 × 3 × 1.5 mm, were carefully prepared on the facial surfaces of each sample. To ensure uniform cavity dimensions, a stainless steel matrix band with a predetermined window was used as the template. The depth was verified using a periodontal probe, and the bur was replaced every five cavity preparations to maintain precision. These cavities were thoroughly cleaned using an air-water syringe, followed by desiccation.

Restorative protocol

**Figure 2:**
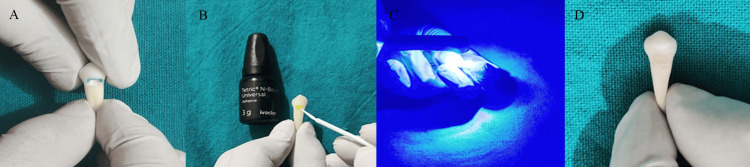
Restorative procedure (A) Acid etching of enamel margin after cavity preparation on the facial aspect of premolar. (B) Application of adhesive bonding agent. (C) Light curing of applied bonding agent. (D) Restoration with nanohybrid composite. The figure is the author's original work of the steps done for the restoration procedure used in the study.

Cavity preparation followed a selective etch-and-rinse bonding protocol. A 37% phosphoric acid gel (Tetric N-Etch, Ivoclar, Schaan, Liechtenstein) was applied for 15 seconds and thoroughly rinsed with water for 20 seconds. The cavity was gently dried with blotting paper to maintain a slight moisture content. A universal bonding agent (Tetric N-Bond Universal, Ivoclar, Schaan, Liechtenstein) was carefully applied, followed by evaporation of the excess solvent with a gentle air stream. The bonding agent was light-cured for 10 seconds using a light-emitting diode (LED) curing unit (3M Elipar DeepCure-S, Saint Paul, MN) at an intensity of 1600 mW/cm². A nanohybrid composite resin (Tetric N-Ceram, Ivoclar, Schaan, Liechtenstein) was incrementally placed into the cavity, with each layer light-cured for 10 seconds according to the manufacturer’s instructions (Figure 2).

All 45 premolars restored with the nanohybrid composite were classified into three groups: group 1 (control) was not exposed to radiation; group 2 (experimental) was exposed to radiation without lead shielding; and group 3 (experimental) was exposed to radiation with lead shielding.

Radiation protocol

**Figure 3:**
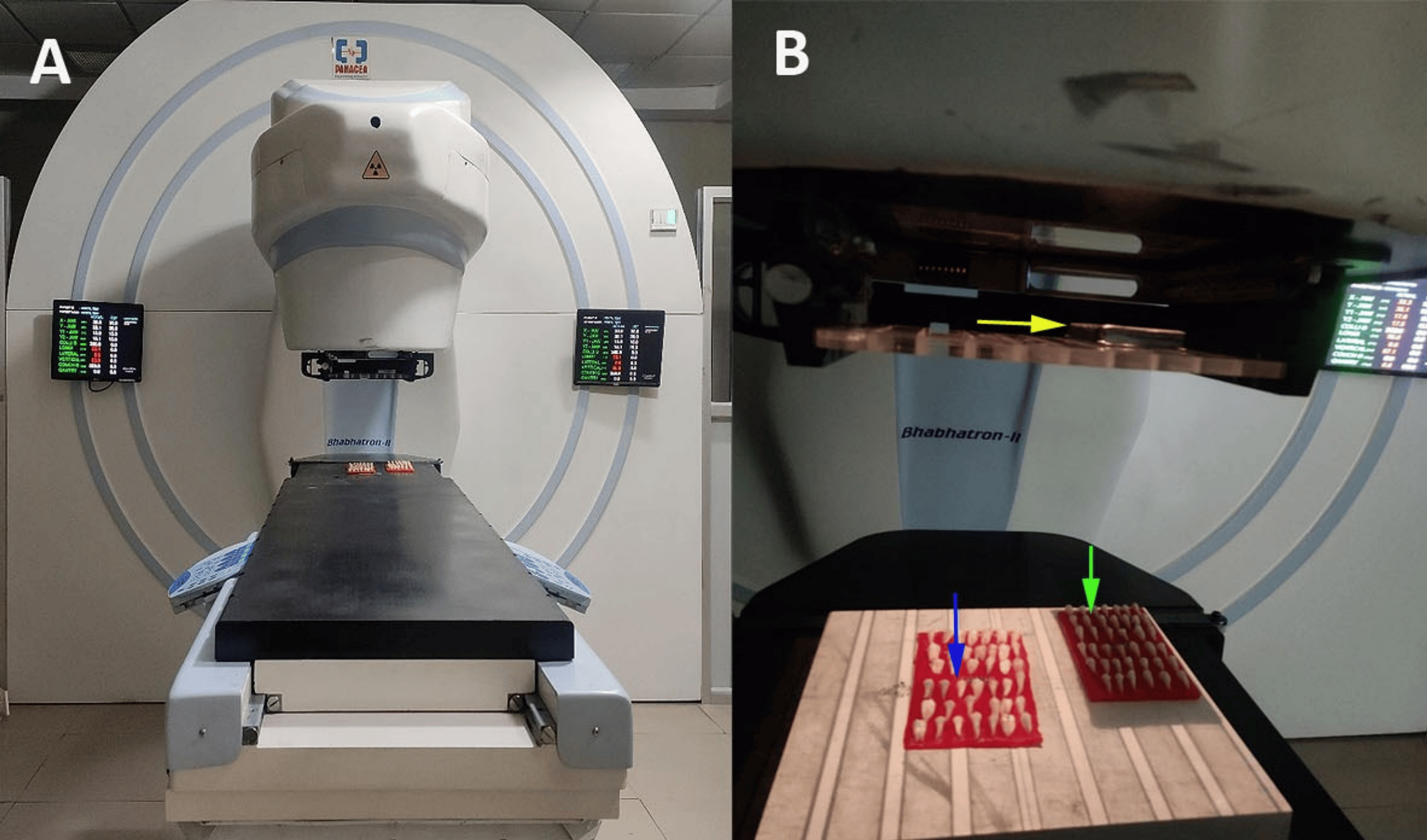
Radiation exposure of cavity-filled teeth. (A) Cobalt-60 teletherapy machine. (B) Machine with lead shield (yellow arrow). Group 1 without lead shield (blue arrow) and group 2 with lead shield (green arrow). The figure is the author's original work where a cobalt-60 teletherapy machine was used for gamma radiation in the study with lead shielding in the collimator.

Gamma radiation therapy was administered using a cobalt-60 teletherapy apparatus (Panacea Medical Technologies Pvt. Ltd., Bangalore, India) in accordance with the prescribed therapeutic dosage for the treatment of head and neck malignancies at Homi Bhabha Cancer Hospital, Sangrur, India by an experienced radio-oncologist. An average energy of approximately 1.25 MeV was delivered by the machine per session. The specimens were irradiated with a cumulative dose of 70 Gy of gamma radiation applied in a fractionated regimen (2 Gy/5 days per week), conducted during daily sessions over a duration of seven weeks. The samples classified within group 3 were protected utilizing 11-mm lead shielding placed in the collimator [14,15]. The samples were stored at 37°C for 24 hours in distilled water. Following the preservation period, the specimens underwent thermocycling (Biotron Healthcare Pvt. Ltd., Mumbai, India) at temperature intervals ranging from 5°C to 55°C, with a dwell time of 15 seconds and a transfer time of one minute for a total of 500 cycles (Figure 3).

Assessment of microleakage

**Figure 4:**
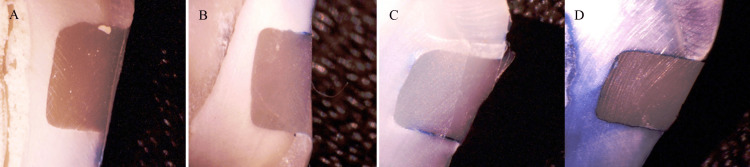
Dye infiltration scoring. (A) No sign of dye infiltration. (B) Dye infiltration extending to the enamel. (C) Dye infiltration at the cavity margin extending into the dentin. (D) Dye infiltration at the cavity margin reaching the axial wall. The figure is the author's original work of the stereomicroscopic images at 20x magnification to assess dye infiltration.

The extent of the microleakage was assessed using the dye penetration method. To isolate the restoration, the apical region of each specimen was coated with sticky wax, and all surfaces except the restoration and a 1 mm surrounding margin were covered with two coats of transparent nail varnish. The teeth were then immersed in a 1% methylene blue dye solution for 24 hours, after which they were thoroughly rinsed and stripped of the wax. Each specimen was sectioned faciolingually through the center of the restoration using a micromotor handpiece equipped with a diamond disc. The dye penetration was examined under a stereomicroscope at 20× magnification (Biotron Healthcare, Mumbai, India) to evaluate the degree of microleakage using a grading system as follows: score 0, absence of dye infiltration; score 1, dye has reached the enamel or cementum at the cavity margin; score 2, dye has infiltrated the cavity margin into the dentin; and score 3, dye penetration at the cavity margin has progressed to the axial wall [13]. The dye infiltration technique is notably straightforward, cost-effective, and does not require specialized apparatus beyond the stereomicroscope, which is widely accessible in dental research or clinical environments (Figure 4).

Statistical analysis

Data were collected and analyzed using IBM SPSS Statistics for Windows, version 23.0 (IBM Corp., Armonk, NY). The normality of the data was assessed using the Shapiro-Wilk test, which indicated that the data did not follow a normal distribution. Consequently, non-parametric tests, specifically the Kruskal-Wallis test, were employed to compare the mean scores of dye infiltration. Post-hoc pairwise comparisons were performed using Dunn’s test to further analyze the significant differences between the groups. A chi-square test was performed between groups and dye infiltration score. The level of significance was considered less than 0.05 (p-value < 0.05).

## Results

For group 1, the median dye infiltration score was 1, with a mean value of 0.87 ± 0.83. Group 2 had a median dye infiltration score of 3, with a mean value of 2.47 ± 0.51, and group 3 exhibited a median dye infiltration score of 2, with a mean score value of 2.06 ± 0.50. The Shapiro-Wilk analysis obtained a p-value of less than 0.001 for all groups, indicating non-normality. Hence, non-parametric tests were used to compare study groups (Table 1).

**Table 1:** Mean dye infiltration score values for each group and normality distribution. * P < 0.05: significant for non-normal distribution of data. Data are presented in the form of mean and standard deviation (SD).

Group	N	Median	Mean	SD	Shapiro-Wilk	P-value of Shapiro-Wilk
Group 1	15	1	0.87	0.83	0.79	0.004*
Group 2	15	3	2.47	0.51	0.63	0.001*
Group 3	15	2	2.06	0.50	0.64	0.001*

The null hypothesis was rejected because significant differences were observed between the groups. Group 1 had a mean value of 0.87 and a rank of 148, with a statistically significant difference, as indicated by the Kruskal-Wallis test (p = 0.001). Group 2 had a mean of 2.47 and a rank of 460, while group 3 had a mean of 2.06 and a rank of 426. A significant p-value suggests a notable difference between the groups. Group 1, which had the lowest median or mean dye penetration score, along with statistical significance, would likely be considered to have the best result, followed by group 3 and group 2 (Table 2).

**Table 2:** Comparison of mean dye infiltration score by Kruskal-Wallis test. * P < 0.05: significant.

Groups	N	Median	Mean	Rank	p-value
Group 1	15	1	0.87	148	0.001*
Group 2	15	3	2.47	460	
Group 3	15	2	2.06	426	

Furthermore, post-hoc analysis (Dunn test) revealed that when comparing group 1 with each of the other groups (2 and 3), the mean differences were all negative, indicating that group 1 tended to have lower values than the other groups. All these differences were statistically significant, with p-values less than 0.001 (Table 3).

**Table 3:** Pairwise comparison with post-hoc analysis by Dunn test. * P < 0.05: significant; SE: standard error.

Pairwise group		Mean difference	SE	t-value	P-value
Group 1	Group 2	-1.60	0.272	-6.36	0.001*
	Group 3	-1.19	0.272	-5.87	0.001*
Group 2	Group 3	0.41	0.272	0.49	0.988

The chi-square test for the distribution of dye infiltration scores among the three groups yielded a chi-square value of 21.95 with a p-value of 0.0012. The highest proportion of score 3 (complete dye penetration) was observed in group 2 (47%), while group 1 had no cases of score 3. Group 3 had a relatively balanced distribution across score 2 and score 3 (Table 4).

**Table 4:** Frequency distribution of dye infiltration score in study groups. * P < 0.05: significant. Data are presented in the form of n (%).

Groups	Dye infiltration score				Chi stats	P-value
	Score 0	Score 1	Score 2	Score 3		
Group 1	6 (40%)	5 (33%)	4 (27%)	0 (0%)	21.95	0.001*
Group 2	0 (0%)	0 (0%)	8 (53%)	7 (47%)		
Group 3	2 (13%)	1 (7%)	6 (50%)	6 (40%)		

## Discussion

Radiotherapy is frequently employed as a standard treatment modality, often in conjunction with chemotherapy or surgical interventions, and the administered doses typically range between 40 and 70 Gy [6]. In the context of patients diagnosed with cancer who are receiving radiation therapy, the application of resin composite restorations of teeth employing direct bonding methodologies is preferred over the use of metallic restorations, as this approach markedly diminishes the likelihood of radio-mucositis [13]. In a systematic review by Palmier et al. [13], the use of composite adhesive material with fluoride gel was recommended for class V cavities in patients undergoing head and neck radiation therapy. The present study used nanohybrid composites as a restorative material, which was in accordance with a study by Eltohamy et al. [16], who concluded that nanohybrid composites are the material of choice as restorative materials in patients undergoing radiation therapy because they possess superior mechanical properties and color stability.

Naves et al. [17] elucidated that the temporal relationship between restorative interventions and radiotherapy exerts a profound influence on the bonding efficacy to enamel and dentin, thereby affecting the degree of microleakage. The application of gamma radiation markedly reduces the bond strength between human enamel and dentin by obstructing the development of the hybrid layer and amplifying the frequency of cohesive failures within the dental substrate when adhesive restorative procedures are performed after radiotherapy. Conversely, performing restorative procedures prior to irradiation did not yield any statistically significant changes in bond strength. According to de Amorim et al. [18], pre-emptive therapeutic intervention of caries lesions prior to the initiation of radiation therapy is imperative to mitigate disease progression and reduce microbial burden. This anticipatory strategy is vital because ionizing radiation adversely affects the adhesion properties between restorative substances and dental tissues, which may result in compromised restorative outcomes following radiotherapy. Consequently, it is recommended that restorative procedures be performed before radiation exposure. Therefore, the present study used restorative procedures prior to radiotherapy.

The findings of the current study demonstrated that gamma radiation exerted a significant influence on the marginal integrity of restorations, irrespective of the presence of lead shielding. Yoshikawa et al. [8] examined the implications of gamma-ray irradiation, revealing substantial degradation of dentin collagen, positing that such irradiation would diminish the bonding strength of the resin composite to dentin, consequently elevating the likelihood of microleakage. Comparable outcomes have been documented by Bansal et al. [12]. The initiation of matrix metalloproteinases within the dentin matrix as an outcome of radiotherapy may play a role in detachment at the interface between the tooth and restoration, thereby resulting in augmented marginal discrepancies post radiotherapy. Furthermore, radiotherapy exerts a detrimental effect on collagen fibers, which ultimately reduces the bond strength between dentin and composite materials while concurrently impairing marginal adaptation [19].

The present study used the extraoral method of lead shielding, where an 11-mm lead shield was placed in the collimator, compared to placing the lead shield around the teeth samples, as used in a study by Gupta et al. [10] where 0.5-mm lead shield was used around the teeth and found that it was effective in reducing microleakage. However, this method only reduces scattering to the protected area and does not reduce the effects of the primary beam [20]. In contrast, collimator shielding is better for overall protection and field shaping. This method effectively reduces the radiation effects from the primary beam; therefore, we used this method in our study. The radiation protection by lead shielding depends on the half-value layer (HVL) and tenth-value layer (TVL) [14]. Previous studies have reported that increasing the thickness of lead shielding to more than 5 mm would reduce the radiation dose by 90% [21]. The magnitude of the dose administration is inversely related to the square of the separation from the radiation source (inverse square law). Consequently, increasing the spatial separation of the unaffected mucosal surface from the radiation beam diminishes its response. Acrylic resin, when accompanied by a lead shield of 2 mm in thickness, has been documented to reduce the dose received by normal tissues by 20% and 15% for cobalt 60 MeV and 6 MeV X photon radiation, respectively [22]. A lead shield with a thickness of 11 mm is deemed optimal and applicable for shielding against high-energy gamma radiation (364 keV). Lead shields with thicknesses varying from 11 to 28 mm exhibit an attenuation efficiency ranging from 90.6% to 99.0% [23]. Therefore, the present study used an 11-mm lead shield in a collimator.

The results of the present study indicate that although lead shielding was effective in reducing microleakage compared to the group without lead shielding, the results were not statistically significant. This contradicts the findings of Gupta et al. [10], where a 0.5-mm lead shield was found to be effective in reducing microleakage. This disparity in results could have been due to the fact that when lead is positioned in direct contact with the teeth, it effectively attenuates radiation prior to its interaction with the enamel and dentin, thereby mitigating radiation exposure to dental tissues. If the protective shield is situated at a distance, a portion of the scattered radiation may still impinge on the teeth, thereby compromising the efficacy of the shielding.

Clinical implications of the study

The findings of this study highlight the need for an optimized shielding approach in head and neck cancer radiotherapy to minimize radiation-induced dental damage. While collimator-based shielding effectively reduces primary beam exposure, intraoral shielding provides direct protection against scattered radiation, reducing microleakage, and preserving enamel and dentin integrity. A combined approach using both a collimator and intraoral shielding offers the most effective protection, ensuring better radiation attenuation while minimizing secondary effects. Clinicians should prioritize pre-radiotherapy dental restorations and adopt dual-shielding techniques to enhance oral tissue preservation, ultimately improving long-term oral health outcomes in patients undergoing radiotherapy.

Limitations of the study

This study did not evaluate the long-term effects of radiation on restorative materials and dental structures. The sample size was limited, and the clinical conditions may differ from those in controlled experimental settings. Additionally, variations in radiation dose, shielding placement, and patient-specific factors could influence the outcomes, requiring further research for broader clinical validation. Clinical exposure to radiation is associated with detrimental consequences, including reduced salivation, diminished saliva buffering capacity, altered concentrations of salivary electrolytes, and notable alterations in oral microbial communities. Collectively, these elements hinder the remineralization potential of saliva, culminating in radiation-induced caries. Therefore, we advocate for further empirical validation through in vivo investigations, which should encompass additional parameters not explored in our in vitro model, to yield a comprehensive insight into the effects of radiation exposure on oral health.

## Conclusions

It can be deduced from the current investigation that under typical conditions, devoid of any prior radiation exposure in any phase, the nanohybrid composites exhibited markedly superior performance with respect to minimal microleakage. Microleakage, manifesting to varying extents, was identified across all the samples, corresponding to three distinct clinical scenarios. Gamma radiation adversely affects the adhesion between composite substances and dental hard tissues. The implementation of collimator lead shielding did not yield a statistically significant reduction in microleakage when compared with scenarios devoid of lead shielding.
